# A reliable mouse model for studying intestinal epithelial damage

**Published:** 2013-11

Inflammation of the intestinal epithelium is a common feature of many acute and chronic gastrointestinal disorders, including Crohn’s disease. Damage to the intestinal lining is thought to be mediated by increased shedding of epithelial cells and enhanced gut permeability. These processes remain poorly understood at the molecular level, in part because of a lack of suitable animal models. Now, Mark Pritchard and colleagues have established a robust model for examining intestinal epithelial cell shedding, using lipopolysaccharide (LPS) treatment to induce intestinal injury in mice. They show that LPS-treated mice develop diarrhoea, which correlates with intestinal epithelial cell shedding and caspase-3-driven apoptosis. By examining the effects of LPS in knockout mice, they demonstrate a role for TNFR1 and NFκB2 signalling in these molecular events. The model provides a powerful tool for investigating the mechanisms underlying intestinal epithelial damage, which could guide the development of new therapies for gastrointestinal disorders. **Page 1388**

